# Diagnostic biomarkers in ovarian cancer: advances beyond CA125 and HE4

**DOI:** 10.1177/17588359241233225

**Published:** 2024-02-29

**Authors:** Aruni Ghose, Lucy McCann, Shania Makker, Uma Mukherjee, Sri Vidya Niharika Gullapalli, Jayaraj Erekkath, Stephanie Shih, Ishika Mahajan, Elisabet Sanchez, Mario Uccello, Michele Moschetta, Sola Adeleke, Stergios Boussios

**Affiliations:** Department of Medical Oncology, Barts Cancer Centre, St. Bartholomew’s Hospital, Barts Health NHS Trust, London, UK; Department of General Medicine, Newham University Hospital, Barts Health NHS Trust, London, UK; Department of Medical Oncology, Medway NHS Foundation Trust, Gillingham, UK; Department of Medical Oncology, Mount Vernon Cancer Centre, East and North Hertfordshire NHS Trust, London, UK; Department of General Medicine, Newham University Hospital, Barts Health NHS Trust, London, UK; Faculty of Epidemiology and Population Health, London School of Hygiene and Tropical Medicine, London, UK; Barts and the London School of Medicine and Dentistry, Queen Mary University of London, London, UK; University College London Cancer Institute, London, UK; Department of Medical Oncology, Barts Cancer Centre, St. Bartholomew’s Hospital, Barts Health NHS Trust, London, UK; University College London Cancer Institute, London, UK; School of Biosciences Education, Faculty of Life Sciences and Medicine, King’s College London, London, UK; Department of Medical Oncology, Northern Ireland Cancer Centre, Belfast City Hospital, Belfast Health and Social Care Trust, Belfast, UK; Department of General Medicine, Newham University Hospital, Barts Health NHS Trust, London, UK; Department of Acute Medicine, Lincoln County Hospital, United Lincolnshire Hospitals NHS Trust, Lincoln, Lincolnshire, UK; Department of Medical Oncology, Apollo Cancer Centre, Chennai, Tamil Nadu, India; Department of Medical Oncology, Medway NHS Foundation Trust, Gillingham, UK; Department of Medical Oncology, Southampton General Hospital, University Hospital Southampton NHS Foundation Trust, Southampton, UK; Novartis Institutes for Biomedical Research, Basel, Switzerland; Department of Clinical Oncology, Cancer Centre at Guy’s, Guy’s and St. Thomas’ NHS Foundation Trust, London, UK; Faculty of Life Sciences and Medicine, School of Cancer and Pharmaceutical Sciences, King’s College London, Guy’s Campus, London, WC2R 2LS, UK; Department of Medical Oncology, Medway NHS Foundation Trust, Gillingham, UK; Faculty of Life Sciences and Medicine, School of Cancer and Pharmaceutical Sciences, King’s College London, London, UK; Kent and Medway Medical School, University of Kent, Canterbury, UK; AELIA Organization, Thermi, Thessaloniki, Greece

**Keywords:** circulating tumour biomarkers, diagnostic biomarkers, molecular testing, ovarian cancer, tumour biomarkers

## Abstract

Ovarian cancer (OC) is the most lethal gynaecologic malignancy, attributed to its insidious growth, non-specific symptoms and late presentation. Unfortunately, current screening modalities are inadequate at detecting OC and many lack the appropriate specificity and sensitivity that is desired from a screening test. Nearly 70% of cases are diagnosed at stage III or IV with poor 5-year overall survival. Therefore, the development of a sensitive and specific biomarker for early diagnosis and screening for OC is of utmost importance. Currently, diagnosis is guided by CA125, the patient’s menopausal status and imaging features on ultrasound scan. However, emerging evidence suggests that a combination of CA125 and HE4 (another serum biomarker) and patient characteristics in a multivariate index assay may provide a higher specificity and sensitivity than either CA125 and HE4 alone in the early detection of OC. Other attempts at combining various serum biomarkers into one multivariate index assay such as OVA1, ROMA and Overa have all shown promise. However, significant barriers exist before these biomarkers can be implemented in clinical practice. This article aims to provide an up-to-date review of potential biomarkers for screening and early diagnosis of OC which may have the potential to transform its diagnostic landscape.

## Introduction

Ovarian cancer (OC) is a gynaecologic malignancy that forms *via* two different carcinogenic mechanisms. These are subsequently classified into two types based on the site of origin. Type I OC tends to be comparatively more genetically stable and develop from known precursor lesions. By contrast, type II OC tends to be high-grade serous carcinomas which are aggressive tumours derived from fimbriae of the fallopian tube.^1^ OC is commonly present late in the disease course due to its latent symptoms and insidious onset. Around 60% of patients have International Federation of Gynaecology and Obstetrics (FIGO) stage III–IV disease at initial diagnosis and this is associated with 5-year survival rates of 27% and 13%, respectively. Only 30–40% of patients have FIGO stage I–II at diagnosis, which is associated with a 5-year survival rate of more than 80%. Altogether, this makes it the fifth and sixth most common cause of cancer-related death in the United States and the United Kingdom, respectively, and the gynaecologic malignancy with the worst prognosis overall.^2–4^

There is a lack of effective screening modalities in the diagnosis of OC. The current early diagnostic method includes a three-staged evaluation using tumour marker assays, ultrasound technology and the patient’s menopausal status. Early detection is a significant challenge as women may not seek medical attention early enough in their disease course due to latent symptoms, with up to 20% of cases being missed altogether.^5^ Moreover, there are germline pathogenic variants in BRCA genes which are detected in around 6–15% of diagnosed epithelial OC patients. Women identified as having *BRCA1* variants have a 13–60% increased lifetime risk and those with *BRCA2* variants have a 10–25% increased lifetime risk of developing epithelial OC. This suggests a potential for screening for OC in women with known BRCA mutations.^6^ There is clearly an unmet need for identifying a biomarker that is sensitive and specific enough to detect OC and have a meaningful impact on identifying cases early enough to positively influence survival course.^7^ This review article explores the wide variety of biomarkers that may contribute towards the diagnosis of OC.

## Serum biomarkers

Over the last 40 years, a number of biomarkers have been investigated to advance the clinical care of OC. Some of the other biomarkers explored include those from urine, cervical smears and oral swabs. None of these have shown any real potential for clinical translation.^8^

### CA 125

The Carbohydrate Antigen 125 (CA 125) is the first and most widely recognized serum biomarker, approved by the US Food and Drug Administration (FDA) in 1981 for the detection of OC.^9^ However, its contribution towards screening is less certain. It is a glycoprotein that is present in most normal gynaecological tissue but is also elevated in benign conditions and non-ovarian malignancies.^10,11^ Its levels are undetectable or modest in some OC subtypes with one in five epithelial cancers expressing undetectable levels of CA125,^12^ whilst in patients with stage I carcinomas, its sensitivity is even lower at 23–50%.^13^ The Prostate, Lung, Colorectal and Ovarian (PLCO) Cancer Screening Trial, run by the National Cancer Institute in 1993, looked at over 75,000 women who were randomized to receive either annual screening with serum CA125 measurement and transvaginal sonography or the non-intervention arm. No difference in disease-specific mortality was found between the two groups.^14^ Furthermore, over-diagnosis from false-positive results led to both mild (bleeding, bruising, nausea) and occasionally severe complications in participants due to the screening and subsequent diagnostic procedures. The UK Collaborative Trial of Ovarian Cancer Screening (UKCTOCS) assessed 200,000 women followed up for an average of 16 years, again finding no difference in mortality in those who were screened *versus* those who received no screening.^15^

### Human epididymis protein 4 (HE4)

In 2008, the FDA approved HE4 as another glycoprotein biomarker for monitoring recurrence or progressive disease, as it is seen to be upregulated in most, but not all, epithelial ovarian cancers (EOC).^16^ It is upregulated by various non-ovarian factors such as age, menstrual cycle, smoking, renal failure and other malignancies, particularly those of reproductive origin but also respiratory cancers.^17^ As HE4 increases significantly with age and menopausal status, different reference values are used for pre- and postmenopausal women, with a higher threshold in menopausal women.^18^ Unlike CA125, HE4 is better at differentiating EOC from other benign ovarian masses such as benign tumours.^19^ While on its own, its use as an early detection tool is limited but in combination with other biomarkers, it could play an important role though this has to be validated in prospective studies.

### Combined detection of CA125 and HE4

The relative merits of CA125 and HE4 are a topic of ongoing debate. Whilst various meta-analyses have concluded similar sensitivity between the two biomarkers (approximately 0.75), most studies show a more favourable specificity of HE4 compared to CA125 (approximately 0.90 *versus* 0.83).^20^ Despite the favourable diagnostic performance, the use of these biomarkers in isolation could miss a large proportion of patients.

In an attempt to find novel and more clinically valuable serum biomarkers, Mukama *et al.* performed a case–control study to assess the presence of biomarkers in women with OC. From 92 biomarkers tested, nine showed discriminatory potential: CA125, HE4, FOLR1, KLK11, WISP1, MDK, CXCL13, MSLN and ADAM8. Unfortunately, none appeared to increase the diagnostic power of CA125 alone and are therefore unlikely to be clinically meaningful in further research.^21^

## Multivariate index assay

Multivariate index (MVI) assays have been developed to mitigate the limitations of single serum biomarkers in EOC, especially during the pre-surgical evaluation of adnexal masses. These assays combine serum biomarker levels with other factors such as patient age, metabolite level, menopausal status and ultrasound results to improve the effectiveness of patient triage and ultimately patient outcomes. Some of these assays are described here.

### Risk of malignancy index

In 1990, the first MVI assay was proposed – the risk of malignancy index (RMI). This combines three pre-surgical factors to produce a triage score: serum CA125 level, menopausal status and abdominal or transvaginal ultrasound score. Studies have demonstrated that RMI has the potential to improve the sensitivity and specificity of serum CA125 alone by up to 85.4% and 96.9%, respectively.^22^ Since its discovery, the tool has been integrated into clinical practice for risk stratification of those presenting with pelvic masses and/or with symptoms within the United Kingdom and internationally.^23^ However, the exact parameters for each criterion have been updated since its development and a systematic review has suggested the superiority of other ultrasound models, such as the International Ovarian Tumour Analysis.^24^

### OVA1

The Ova1 Multivariate Index Assay (MIA) was the first diagnostic MIA to combine CA125, with four other serum biomarkers: transthyretin, transferrin, apolipoprotein A1 and β-2 microglobulin, alongside imaging results and menopausal status. It has been shown to detect 76% of malignancies that would be otherwise missed by the use of CA125 alone.^25,26^ It has a higher sensitivity than CA125 alone (94% *versus* 77%) but a lower specificity (54% *versus* 94%). It was the first MIA assay to receive FDA approval. Rather than being used as a diagnostic test, the primary purpose of Ova1 is as a triage test for primary care physicians when considering referral to gynaecological oncology in women with suspected pelvic masses requiring surgery.^27,28^ The test is carried out once imaging has confirmed the presence of a mass and that the mass has been established to require surgery. The high false-positive rate of Ova1 is recognized as a major limitation.^29^

### ROMA

The Risk of malignancy algorithm (ROMA) integrates menopausal status, CA125 and HE4 concentrations to diagnose women with a pelvic mass. This dual biomarker approach has shown high performance in meta-analyses^30,31^ and received FDA approval in 2011.^32^

When compared to RMI, ROMA had a higher sensitivity (95% *versus* 85%) and comparable rates of specificity. The ability of ROMA to detect early disease compared to RMI was even more pronounced at 85% *versus* 65%. When compared to OVA1, ROMA has a higher sensitivity (97% *versus* 87%) and OVA1 showed a higher specificity (83% *versus* 55%).^33^

### Overa

The Overa test is a second-generation MIA (MIA2G), which consists of CA125, HE4, apolipoprotein A1, follicle-stimulating hormone and transferrin. A multicentre study by Coleman *et al.* demonstrated superior specificity (69% *versus* 54%) and positive predictive value (40% *versus* 31%) of MIA2G over MIA while maintaining similar sensitivity and negative predictive value.^34^ Another multicentre study by Shulman *et al.* compared Overa with ROMA in 245 patients and found that whilst Overa had a lower rate of misclassification as compared to ROMA (22 *versus* 51), ROMA had a lower frequency of early-stage misclassification (8 *versus* 22).^35^ Overa received FDA approval in 2016 to assess the risk of OC in women who present with pelvic masses and are planned for surgery.^36^ It is not to be used as a screening test.^37^

### Other MVIs

Recent research has focused on evaluating different MIAs with various combinations of biomarkers using immunoassays. Simmons *et al.* found that the MIA using CA125, HE4, MMP-7 and CA72-4 had an extremely high specificity of 98% and sensitivity of 83.2%.^38^ In Muinao *et al.*’s review, the use of CA-125 with EGFR, CA 19-9, G-CSF, Eotaxin, IL-2R, cVCAM and MIF had the highest specificity (98.7%) and sensitivity (98.2%) compared to all other analysed MIAs.^39^

The adnexal mass risk assessment (AMRA) was another MVI designed to stratify suspected OC patients into three risk groups (low, intermediate and high) necessitating varying surgical urgency. Its seven biomarkers included CA125, HE4, Apo A1, HE4, transferrin, transthyretin and β-2 microglobulin. Zhang *et al.* used retrospective data which revealed better diagnostic outcomes with AMRA than that of CA125 across various histological subtypes of OC and detection of early-stage disease.^40^ This inspired the OVAnex study for the prospective validation of AMRA’s sensitivity, specificity, PPV and NPV (NCT04487405). These studies indicate the potential to identify a new MVI for the diagnostic landscape in OC, with an emphasis on maximizing specificity and sensitivity to aid in early detection. However, despite advancements in prediction models, there currently remains no national screening or diagnostic consensus for preoperative evaluation of adnexal masses.

## *VEGF* (angiogenesis-related biomarkers)

Angiogenesis involves the development of new vessels *via* stimulation of vascular endothelial cells and is a main component of tumour progression in solid tumours, including OC.^41^

The most important biomarker identified in the process of angiogenesis is VEGF. The VEGF family comprises VEGF-A, B, C, D and PlGF (placental growth factor) proteins. Their receptors for signalling include VEGFR 1, 2 and 3. VEGF-A binds to VEGFR1, 2 to facilitate tumour dissemination while VEGF-C, D binds to VEGFR3 to stimulate lymphangiogenesis.^42,43^ There is a notable difference in expression levels in distant ovarian metastases with one study finding higher expression levels of VEGF-A (*p* = 0.022), D (*p* = 0.010) and VEGFR1 (*p* = 0.046) in distant metastases (omental) as compared to primary high-grade serous ovarian cancer (HGSOC) lesions.^42^ Another study however showed that VEGF-A values were lower in more advanced disease stages, while CA125 levels showed an upward trend.^43^

Several studies have explored the discriminatory potential of VEGF to diagnose OC compared to CA125 and HE4. The earliest study examined 100 OC cases and 130 controls, which included benign ovarian tumours and healthy individuals. VEGF, CA125 and HE4 were significantly raised in OC cases than within control subjects. CA125 used alone had the highest sensitivity but all three biomarkers in combination showed higher levels of sensitivity (84% in stage I, 96% in stage II-IV). Diagnostic accuracy as inferred from the area under ROC curves (AUC) showed a similar trend; it was highest when CA125 was used alone but was more for the triple marker combination. Such results suggested that these biomarkers could have a role in early detection.^44^ A meta-analysis using 10 studies and 1331 subjects further proved moderate diagnostic accuracy of VEGF in OC with a pooled sensitivity and specificity of 67% and 78%, respectively.^45^

### Osteopontin

Osteopontin (OPN) is an extracellular matrix glycoprotein, which functions as a cell adhesion protein and has cytokine properties. Its role in wound healing, inflammation, macrophage regulation and tumour dissemination is well documented.^46^ In terms of its role as a diagnostic biomarker, one meta-analysis analysed 15 case–control studies, containing a total of 1653 subjects and found a statistically significant positive correlation between elevated OPN levels and OC.^47^ Hu *et al.* conducted another meta-analysis, which showed an overall sensitivity and specificity of 66% and 88%, respectively, of OPN alone for diagnosis, which showed OPN to be a useful biomarker with convincing accuracy.^48^ However, a comparison was not made with CA-125, therefore rendering its diagnostic prowess over this traditional biomarker relatively unknown. Varying levels of OPN expression across different histologic subtypes were noted. For example, there were higher levels of mucinous compared to serous OC subtypes. Levels were also relatively higher in more advanced stages of disease.^47,48^ Studies have shown OPNc isoform is linked to chemotherapy resistance in cell models and knockdown of OPNc increases sensitivity to therapy.^49^ This OPNc characteristic could be used to explore aggressive disease cohorts during screening and could potentially guide treatment decisions.

In a study evaluating an angiogenesis multi-marker panel consisting of 16 biomarkers including conventional CA125 and HE4 across 156 patients. They included 50 healthy controls, 38 OC, 6 borderline and 62 non-malignant ovarian masses. It found Osteopontin to be a promising diagnostic single angiogenesis marker whilst comparing OC *versus* non-OC (69% sensitive and 78% specific) as well as malignant *versus* benign ovarian tumours (72% sensitive and 82% specific). The addition of OPN to CA125 and HE4 forming a triple marker panel saw significantly improved accuracy than CA125 or HE4 alone.^50^

### Mesothelin

Mesothelin (MSLN) is a membrane-bound surface glycoprotein secreted by mesothelial cells and is strongly expressed in OC, especially amongst non-mucinous subtypes.^51^ Through binding with CA125, it facilitates cell-to-matrix adhesion and downregulates intercellular adhesion for intra-peritoneal metastasis.^52^ Studies have shown serum levels of mesothelin were raised in OC with higher levels observed in bilateral disease, massive ascites (>2000 ml) and advanced FIGO stage (III–IV).^53,54^ Mesothelin expression levels in urine have been investigated and there are higher levels compared to serum both in the early stage (42% *versus* 12%) and in late-stage disease (75% *versus* 48%).^55^ Using mesothelin in combination with HE4 and CA 125 has been shown to improve sensitivity and specificity.^54,56^

Wu *et al.* studied a splice variant of soluble MSLN called soluble megakaryocyte-potentiating factor (SMRP). In 78 OC, 84 benign ovarian tumours and 58 healthy volunteers, median SRMP values were 3.5, 0.5 and 0.5, respectively. The values differed according to the FIGO stage – 1.16 in early *versus* 4.59 in late-stage disease. The sensitivity and specificity of SRMP, CA125, SRMP and CA125 combined were 82%, 97%, 98% and 97%, 90%, 88%, respectively.^57^ Okla *et al*. comprehensively evaluated and correlated plasma, peritoneal fluid and tumour tissue levels of MSLN in OC. Mean plasma MSLN levels were notably higher in OC^54^ as compared to benign ovarian tumours^12^ and healthy controls.^11^ Significant differences were observed with regard to the FIGO stage (34 in early *versus* 81 in advanced) and grade (41 in low *versus* 73 in high grade). Mean peritoneal fluid MSLN levels showed differences in grade (397 in low *versus* 792 in high). Tumour tissue MSLN expression was 1.9-fold higher in OC *versus* 0.1 in benign tumours and 2.9 in high-grade *versus* 1.7 in low-grade OC. All the aforesaid shared a positive correlation with CA125. Also, plasma MSLN correlated well with peritoneal fluid and tissue counterparts hence deeming it a diagnostically useful biomarker surrogate.^54^

### Interleukins

Interleukin-6 is a proinflammatory cytokine capable of altering the tumour microenvironment, leading to angiogenesis *via* overexpression of VEGF. It also causes neoplastic basement membrane invasion through overexpression of matrix metalloproteinases.^58^ IL-6 has been investigated as a diagnostic tool for screening of suspected ovarian mass. Serum IL-6 levels are higher in advanced HGSOC and when used in combination with other standard markers consisting of CA125, HE4, ROMA and RMI, there was enhanced sensitivity, specificity, diagnostic accuracy, overall predictive probability and lower false positives.^59^

Its levels in ascitic fluid have also been studied. A meta-analysis was conducted looking at the diagnostic utility of IL-6 expression levels in ascitic fluid and serum across 37 studies in almost 7000 subjects. Levels of ascitic IL-6 were significantly higher in late-stage OC compared to early-stage OC and benign controls.^60^ Pooled sensitivity and specificity for IL-6 in serum and ascites were 76%, 72% and 84%, 74%, respectively. While these analyses suggest the potential for the utility of ascitic expression of IL-6 in late-stage OC, its use in early detection still needs to be evaluated.^60^

## MicroRNA biomarkers

Small non-coding microRNAs (miRNA) are short, single-stranded RNA segments (approximately 22 nucleotides long) that bind to complementary messenger RNA (mRNA) sequences and interfere with protein translation.^61^ miRNAs modulate apoptosis, differentiation and cell cycle control, enabling them to function as tumour suppressors or oncogenes.^62^ Importantly, miRNAs are tissue specific and are secreted from cells after biogenesis. miRNAs are detectable in all bodily fluids (e.g. plasma/serum, urine and cerebrospinal fluid), as well as extracellular vesicles (EVs) and the tissue microenvironment. EVs include exosomes, microvesicles or apoptotic bodies and vary in composition. Exosomes only contain a small percentage of circulating miRNAs, whereas the larger vesicles (microvesicles or oncosomes) contain a larger proportion of miRNA in addition to larger RNAs.^63^ It has been hypothesized that some miRNAs are present in the blood due to tumour cell lysis and research has shown that there is a correlation between the progression of EOC and increased cancer-derived exosome circulation.^64^ Several miRNAs may be used as an indicator of EOC, such as the overexpression of exosomal miR-100, miR-21, miR-320, and the under expression of miR-126, miR-93 and miR-223.^65,66^

### miRNA-200 family

The miR-200 family consists of five miRNAs: miR-141, miR-200a, miR-200b, miR-200c and miR-429, with deregulated expression often seen in EOC.^67^ Inhibiting miR-200 microRNAs induces epithelial-mesenchymal transition (EMT) and reduces adhesion through upregulating the E-cadherin transcriptional repressors ZEB1, and SIP1, which may promote metastasis in EOC.^64^ Interestingly, miR-141 and miR-200a can improve the sensitivity of paclitaxel by repressing p38 MAPK *via* a mechanism dependent on reactive oxygen species.^68^

### Limitations of microRNAs

Over the last two decades, significant developments have led to a further understanding of how miRNA might play a role in oncologic hallmarks. miRNAs may have remarkable potential in various aspects of OC prediction. However, further work is needed regarding its characterization as a biomarker. In particular, before miRNAs can be utilized as reliable biomarkers for clinical use, the steps involved in processing samples need to be standardized and the platforms for detecting miRNA in tumours and blood need to be refined.^69^ A single miRNA may not be sufficient as a biomarker; thus, a signature involving the detection of several miRNAs may be more appropriate.^70^

## Liquid biopsy

Liquid biopsies are being developed in clinical trials as a non-invasive, potential screening approach to detect and quantify the presence of cancer cells in peripheral blood. These biomarkers could be in the form of circulating tumour cells (CTCs), circulating tumour DNA (ctDNA) or exosomes that are shed from tumour cells into the circulation.^71^ They are being trialled alongside other biomarkers and gene profiling to assist in the early diagnosis of cancers, including breast, colorectal, lung and prostate cancers. They could also have potential utility in assessing tumour response to systemic anticancer therapy as a proxy measure of minimal residual disease to direct the need for further therapy.^72^

### CTCs in OC

CTCs are whole cells that have shed off from tumour and techniques for efficient isolation and characterization have been developed in the last decade. Whether CTC detection is associated with prognosis in OC remains controversial. Nevertheless, standardized methods to identify CTC have potential significant relevance. Subsequently, circulating biomarkers have been researched to try to improve screening. These involve selecting malignant cells that express epithelial or mesenchymal markers (e.g. EpCAM, cytokeratins, N-cadherin and/or vimentin) and excluding cells that express the haematopoietic cell marker CD45. In OC, the presence of these CTCs could provide useful diagnostic information^72^ (Table 1).

**Table 1.** Various characteristics of CTC studies conducted in EOC (including methods of detection and isolation, genetic markers, detection rates, diagnostic sensitivities and specificities).

Method of isolation	Genetic marker	Method of detection	Detection rate (%)	Sensitivity (%)	Specificity (%)	Year [Reference]
Immunomagnetic (AdnaTest) GA 73.3 & MUC1	HER-2, MUC1, MUC16, EpCAM	Multiplex RT-PCR	19–27	–	–	2011 [Aktas 2011], ref 77
Immunomagnetic (CellSearch) EpCAM	EpCAM, M30, CK8, 18, 19	ICC	44	–	–	2011 [Behbakht 2011], ref 78
Immunomagnetic (CellSearch) EpCAM	EpCAM, CK8, 19, 18	ICC	53.8–60	–	–	2013 [Liu 2013], ref 79
Immunomagnetic beads (CD45)	CK, CEP8	ICC, FISH	76.2	76.2	80	2014 [Ning 2014] ref 80
Immunomagnetic (AdnaTest)EpCAM & MUC1	EpCAM, MUC1, MUC16, ERCCI	Multiplex RT-PCR	14	–	–	2014 [Kuhlmann 2014] ref 76
CAM uptake-cell enrichment	EpCAM, CA-125, CD44, seprase, MUC16 & FAP	ICC/RT-qPCR	100	83	97	2014 [Pearl 2014] ref 81
MetaCell	EpCAM, MUC1, MUC16, KRT18, KRT19	ICC/qPCR	65.2	–	–	2015 [Kolostova 2015] ref 83
CAM uptake-cell enrichment	EpCAM, ESA, CA-125, DPP4	ICC	88.6	83	95.1	2015 [Pearl 2014] ref 81
Adna test OvarianCancerSelect AdnaTestEMT & StemCell Select	19 gene transcripts	Multiplex RT-PCR	30	–	–	2016 [Blassl 2016] ref 74
Immunomagnetic beads (EpCAM, HER2 & MUC1)	EpCAM, HER2, MUC1, WTI, P16, PAXS	Multiplex RT-PCR	90–91	–	–	2016 [Zhu 2016] ref 85
AdnaTest OvarianCancer & EMT-1 Select/Detect	EpCAM, ERCCI, MUC1, MUC16 P13Ka, Akt-2	Multiplex RT-PCR	82	>90	>90	2017 [Chebouti 2017] ref 75
Nanoroughened microfluidic platform	EPcam, TROP-2, EGFR, Vimentin, N-cadherin	ICC	98.1	–	–	2017 [Lee 2017] ref 84
Density gradient centrifugation	EpCAM, EGFR, MUC1, HER2, MECOM, HHLA1	ICC, FISH	7.7–26.5	–	–	2017 [Obermayr 2017] ref 85
Microfluidics plus immunomagnetic beads (EpCAM)	EpCAM, CK3-6H5, panCK	ICC	87	–	–	2017 [Rao 2017] ref 86
Immunomagnetic beads (EpCAM & N-cadherin)	EPcam, N-cadherin, CAD, Vimentin	ICC	90	–	–	2018 [Po 2018] ref 87
Microfluidic Parsortix TM	EpCAM, PPIC, MAL2, LAMB1, SERPINE2, TUSC3	RT-qPCR	70	–	–	2017 [Obermayr 2017] ref 85
Tapered slit filter	CK-9, EpCAM	ICC	57.1–76.7	–	–	2019 [Kim 2019] ref 88

CAM, cell adhesion matrix; CTC, circulating tumour cell; FISH, fluorescence *in situ* hybridization; ICC, immunocytochemistry staining; qPCR, quantitative PCR; RT-PCR, real-time PCR technology.

#### Molecular profiling of CTCs

Molecular profiling of CTCs in OCs has revealed 132 markers of diagnostic importance.^73^ Several genes (*EpCAM*, *WT1*, *MUC16*, *MUC1*, *KRT7*, *KRT18* and *KRT19)* have been identified for their high specificity for CTCs from OC (123). EMT (Vimentin, N-cadherin, Snai2, CD117 and CD146) and stem cell (CD44, ALDH1A1, Oct4 and Nanog) gene transcripts have also been shown to be present in CTCs in OC.^74^ Other EMT-gene transcripts include *PI3Kα, Akt-2* and *Twist*.^75^ These EMT gene transcripts may be implicated in tumour resistance; therefore, their identification can help clinicians to deliver effective, tailored treatment. *ERCC1* (Excision Repair Cross-Complementation Group 1) assists with the repair of DNA-platinum resistance and is known to be a predictor of platinum resistance.^76^ While they have shown promise in predicting treatment resistance, their use in early detection is yet to be fully elucidated.

## Conclusion

In summary, OC is a highly lethal disease, owing to its insidious onset and late detection. Currently, CA125 and HE4 are the only approved biomarkers for use in EOC; however, they are not sufficient for early detection, thus novel diagnostic biomarkers are a necessity. Multiple biomarkers across various platforms have been identified that may have potential as a diagnostic or screening tool in the early detection of OC. Further research is required to better understand these biomarkers and improve their diagnostic accuracy in OC which could help accelerate their translation to clinical practice.

## Declarations

**Ethics approval and consent to participate:** Ethics approval or consent to participate was not required for this work.

**Consent for publication:** Not applicable.

**Availability of data and materials:** Data analysed or presented in this work are publicly available from cited source materials or publications.
